# Is Arsenic Exposure a Risk Factor for Metabolic Syndrome? A Review of the Potential Mechanisms

**DOI:** 10.3389/fendo.2022.878280

**Published:** 2022-05-16

**Authors:** Pablo Pánico, Myrian Velasco, Ana María Salazar, Arturo Picones, Rosa Isela Ortiz-Huidobro, Gabriela Guerrero-Palomo, Manuel Eduardo Salgado-Bernabé, Patricia Ostrosky-Wegman, Marcia Hiriart

**Affiliations:** ^1^ Department of Cognitive Neurosciences, Instituto de Fisiología Celular, Universidad Nacional Autónoma de México, Mexico City, Mexico; ^2^ Department of Genomic Medicine and Environmental Toxicology. Instituto de Investigaciones Biomédicas, Universidad Nacional Autónoma de México, Mexico City, Mexico

**Keywords:** metabolic syndrome, arsenic, beta-cell, insulin resistance, obesity, cardiovascular diseases

## Abstract

Exposure to arsenic in drinking water is a worldwide health problem. This pollutant is associated with increased risk of developing chronic diseases, including metabolic diseases. Metabolic syndrome (MS) is a complex pathology that results from the interaction between environmental and genetic factors. This condition increases the risk of developing type 2 diabetes, cardiovascular diseases, and cancer. The MS includes at least three of the following signs, central obesity, impaired fasting glucose, insulin resistance, dyslipidemias, and hypertension. Here, we summarize the existing evidence of the multiple mechanisms triggered by arsenic to developing the cardinal signs of MS, showing that this pollutant could contribute to the multifactorial origin of this pathology.

**Graphical Abstract:**
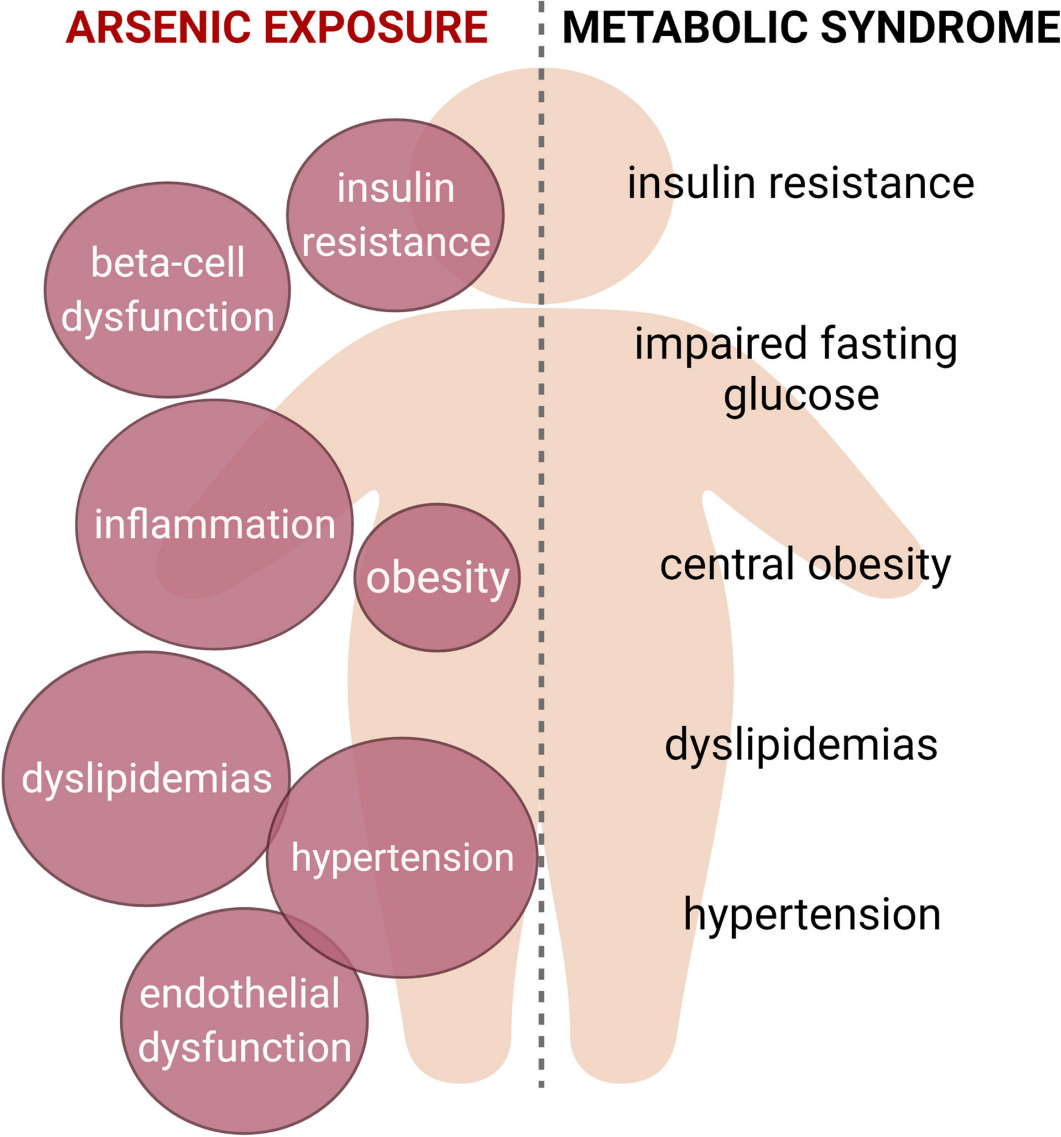
Metabolic syndrome is characterized by the presence of at least three of the following signs: insulin resistance, impaired fasting glucose, central obesity, dyslipidemias and hypertension. Arsenic exposure is linked to the induction of several alterations that could be related with this condition, such as insulin resistance, beta cell dysfunction, chronic inflammation, adipose tissue dysfunction, alterations in serum lipid levels and cardiovascular alterations. Thus, we propose that arsenic should be considered as a risk factor for metabolic syndrome.

## Introduction

Metabolic syndrome (MS) is a multifactorial condition that increases the risk of developing cardiovascular diseases (CVD), type 2 diabetes (T2D), nonalcoholic fatty liver disease (NAFLD), neurodegenerative diseases, and several types of cancer (1–5). The cardinal signs of the MS are 1) abdominal obesity, 2) dyslipidemia (including hypertriglyceridemia and low levels of high-density lipoprotein cholesterol), 3) hypertension, 4) impaired fasting glucose, and 5) insulin resistance (1, 2, 6). At least three of these signs must coexist to be considered MS.

The development of MS has been typically associated with hypercaloric diets, reduced physical activity, and genetic susceptibility. Nevertheless, there is increasing evidence that exposure to environmental pollutants considered as endocrine disruptors contribute to this pathology’s development, including smoking, particulate matter, persistent organic pollutants, and heavy metals, including arsenic (7–10).

Arsenic naturally occurs in the Earth’s crust and is also a pollutant produced by diverse industrial activities such as mining, fracking, coal-fired power plants, arsenic-treated lumber, and arsenic-containing pesticides. This pollutant has been reported in more than 70 countries, including Mexico, the United States, Bangladesh, Chile, Argentina, China, etc (11, 12). Food like rice and seafood might also contain high levels of arsenic (11). Though average environmental arsenic concentrations are low in the soil, air, and water, with mean water concentrations below 1-2 ppb, over 200 million people in multiple regions have underground water with an arsenic concentration above 10 ppb, the upper limit of arsenic concentration in drinking water recommended by the World Health Organization (11, 13). Chronic exposure to low levels of arsenic through drinking water is the primary source of exposure associated with the development of different forms of cancer, T2D, cardiovascular diseases and MS (11). Exposure to arsenic has been linked with MS in several populations (14–16). Still, the precise mechanisms that are triggered by arsenic to contribute to the development of MS remain poorly explored. Thus, this review aims to integrate the known mechanisms that are induced by arsenic that could relate to the hallmark signs of MS.

Arsenic can be present in organic and inorganic compounds. Inorganic arsenic compounds in polluted water are principally trivalent arsenite and pentavalent arsenate. Importantly, arsenite is more cytotoxic than arsenate (11). Nevertheless, the enzymes arsenate reductase and purine nucleoside phosphorylase, during metabolism, reduce arsenate into arsenite (17, 18). Thus, both arsenic species coexist inside the body at any given time, independently of the arsenical ingested. Arsenic metabolism occurs principally in the liver, although some arsenic metabolism can be achieved by other tissues (18). This process starts after entering inorganic arsenic into hepatocytes by aquaglyceroporin 7/9. First, arsenate is reduced to arsenite through the activity of the enzyme arsenate reductase (17, 18). Then, the arsenite methyltransferase (AS3MT) enzyme catalyzes the oxidative methylation of arsenite into pentavalent monomethyl arsenate (MMA^V^), which is reduced to trivalent monomethyl arsenite (MMA^III^) by the enzyme glutathione sulfhydryl transferase omega 1 (GSTO1) (17). Monomethylated arsenicals are further methylated by AS3MT and GSTO1, generating dimethyl arsenate (DMA^V^) and dimethyl arsenite (DMA^III^) (17). The dimethylated arsenicals are excreted into the circulation, delivered to the kidney, and the major arsenic compounds are excreted in urine (17). Intriguingly, some evidence suggests that the methylated forms of arsenic produced during these metabolic pathways are more toxic than the inorganic arsenic by itself (18–21). Also, this complex metabolic processing implies that in exposed humans and animal models, target tissues and cells are exposed to a complex mixture of inorganic and organic arsenicals, which can influence the final effects observed in physiological processes.

Arsenic toxicity has been evaluated in humans drinking arsenic polluted water, in cell cultures and in rodent models. Nevertheless, it has been demonstrated that animal models have several limitations for assessment of arsenic toxicity and its extrapolation to humans. These limitations include arsenic sequestration by the hemoglobin in erythrocytes from rats, and differences in the ratios of DMA/MMA/inorganic arsenic excreted in urine (11, 18). Nevertheless, both rats and mice have AS3MT and a certain degree of arsenic methylation capacity (18). One of the main challenges to effectively integrate the existing data of arsenic effects on the different mechanisms related with the MS is the different experimental approaches. For instance, most *in vitro* studies report arsenic concentrations as µM. However, some studies administrate arsenic in animal models through gavage, calculating the doses as mg/kg of body weight per day. As these doses cannot be converted to mg of arsenic per litre, we keep these as mg/kg. On the other hand, other studies administrate arsenic through drinking water, reporting the doses as mg/L, ppb or ppm. We summarize the information from the animal studies as mg/kg of body weight or as ppb (or ppm according to the magnitude of the doses used), as we consider these units easier to contextualize with the units used in most population-based studies.

## Arsenic as an Obesogenic Pollutant

Arsenic is considered a significant risk factor for developing type 2 diabetes (T2D), but recent attention has focused on its obesogenic effects. Although the epidemiological relationship between chronic arsenic exposure and the development of obesity has not been directly evaluated, an increase in the body mass index (BMI) is observed in individuals living in areas endemic with high concentrations of arsenic in water (22–25). In addition, another study showed that there could be a synergistic relationship between arsenic and BMI to increase the risk of developing T2D (24). Controversially, several epidemiological studies have found no significant association between BMI and arsenic exposure (26–29). In this context, epidemiological studies on the association between arsenic exposure and obesity are quite inconsistent. This could be related to the differences in the levels of arsenic exposure or even in the differences in methods to quantitate arsenic exposure (30). Other factors may also influence the results such as age, gender, dietary factors, study design and population studied.

Evidence on animal models indicates that exposure to inorganic arsenic during adulthood or *in utero* affects body weight. In adult C57BL/6 male mice, exposure to 25 or 50 ppm of sodium arsenite through drinking water for eight weeks induced glucose intolerance without changes in body weight (31). Interaction between treatment with 25 or 50 ppm of sodium arsenite for 20 weeks and high-fat diet (HFD) intake in C57BL/6 male mice resulted in a less weight gain, decreased adiposity, and increased glucose intolerance, compared with mice only exposed to HFD (32). On the contrary, another study in C57BL/6J male mice fed with HFD and exposed to 50 ppm of sodium arsenite for 16 weeks found that arsenic did not significantly affect the final body mass induced by diet, but arsenic reduced adiposity (33). Of note, these studies used relatively high arsenic concentrations, so studies with environmentally relevant arsenic doses could be useful in elucidating the role of arsenic exposure during adulthood in the development of obesity. This is particularly relevant; as many endocrine-disrupting chemicals have non-monotonic dose responses, characterized by a curve whose slope changes direction within a dose range (34). Addressing this hypothesis in future studies by evaluating the effects of a wider range of arsenic doses on the body weight, adiposity, and lean mass in different adult animal models will allow confirming the participation of arsenic exposure during adulthood on obesity development

Arsenic can cross the placental barrier and reach the fetus, causing several epigenetic and physiological changes (35). Because the intrauterine environment is a significant risk factor for obesity in the offspring during adulthood (36), some studies have addressed the possibility of arsenic exposure *in utero* to contribute to obesity in the offspring. In male mice exposed to 100 ppb of sodium arsenite *in utero* and after birth, along with a western-style diet (containing 40.1% fat, 15.5% protein and 44.4% carbohydrates), there were an increase in body weight, liver weight, and serum cholesterol levels (37). In the same way, female CD-1 mice offspring from dams treated with 10 and 42.5 ppm of sodium arsenite during gestation showed increased body weight, adiposity, hyper-leptinemia, and hyper-insulinemia during adulthood (38). Therefore, arsenic exposure could contribute to fetal programming and increase the risk of developing obesity during adulthood.

Although the possible mechanisms of arsenic as an obesogenic agent are not entirely understood, it could also influence adipose tissue dysfunction. Adipose tissue is a complex organ with several normal body physiology and energy homeostasis functions. In the context of metabolic health and diseases, the functions of adipose tissue include nutrient storage in the form of triglycerides, and secreting several factors known as adipokines, which comprise hormones, cytokines, and metabolites that contribute to whole body metabolism through inter-organ communication (39). According to the gene expression patterns and metabolic phenotype of adipocytes, adipose tissue can be categorized as white adipose tissue (WAT) and brown adipose tissue (BAT) (39). During obesity, WAT expands, mainly through adipocyte hypertrophy. Adipose tissue hypertrophya is accompanied by dysfunction, characterized by deregulated lipolysis and lipogenesis, insulin resistance, a proinflammatory state and alteration of adipokine secretion (39). Leptin and adiponectin are two of the most studied adipokines secreted exclusively by adipocytes. These molecules participate in systemic energy balance, regulating appetite signals in the central nervous system, systemic insulin sensitivity and metabolic activity in peripheral tissues (40). The levels and secretion of leptin and adiponectin were decreased by sodium arsenite alone (1 µM for 72 h *in vitro*) or in combination with palmitate (200 µM) when the treatment occurred during adipogenic differentiation of 3T3-L1 cells, but not by treating fully differentiated 3T3-L1 adipocytes, indicating that the effect depends on the differentiation window of the adipocyte lineage at which exposure began (41). Perilipin is a protein associated to the surface of lipid droplets, acting as a barrier that prevents lipase access to triglycerides, regulating lipolysis in the adipocytes. Mice exposed to 100 ppb of sodium arsenite through drinking water for five weeks developed adipocyte hypertrophy, decreased perilipin expression in adipose tissue, and ectopic fat accumulation in skeletal muscle (42). Indicating alterations in lipid homeostasis in the adipose tissue.

BAT are highly specialized fat depots that dissipate storage energy in the form of heat. This effect is due to the high density of mitochondria and the expression of uncoupling protein-1, which uncouples the H^+^ gradient in the inner mitochondrial membrane, producing heat (39). In mice administered with 5 and 10 mg/kg of arsenite by gavage resulted in the impairment of brown adipose tissue differentiation, suppression of lipogenesis, mitochondrial biogenesis, and thermogenesis (43).

Available data suggest that the risk of diseases related to arsenic might be higher in people with increased body weight or BMI. Although the results are highly variable in animal models, evidence indicates that arsenic could impair adipose tissue physiology and cause changes in body weight and adiposity depending on diet, arsenic concentration, and the age of exposure. While most evidence suggest that arsenic is not necessarily a pollutant directly related with adipose tissue expansion and obesity, it is tempting to think that it could induce adipose tissue dysfunction even in the absence of obesity. This could be particularly important as adipose tissue can influence insulin sensitivity and metabolism in other organs, as well as regulate satiety in the central nervous system. Further studies are needed in order to understand whether arsenic can alter inter-organ communication between adipose tissue and other organs important in energy homeostasis.

## The Effects of Arsenic on Beta-Cell Dysfunction

Pancreatic beta-cells respond to elevated plasma glucose levels secreting insulin. This hormone increases glucose disposal by peripheral tissues, reducing glucose levels and decreasing insulin secretion. This process maintains glucose homeostasis in mammals (44), and it is disrupted in the MS, leading to pancreatic beta-cell exhaustion and decreased insulin secretion through poorly unknown mechanisms. Thus, contributing to the development of T2D (45).

**Figure 1:**
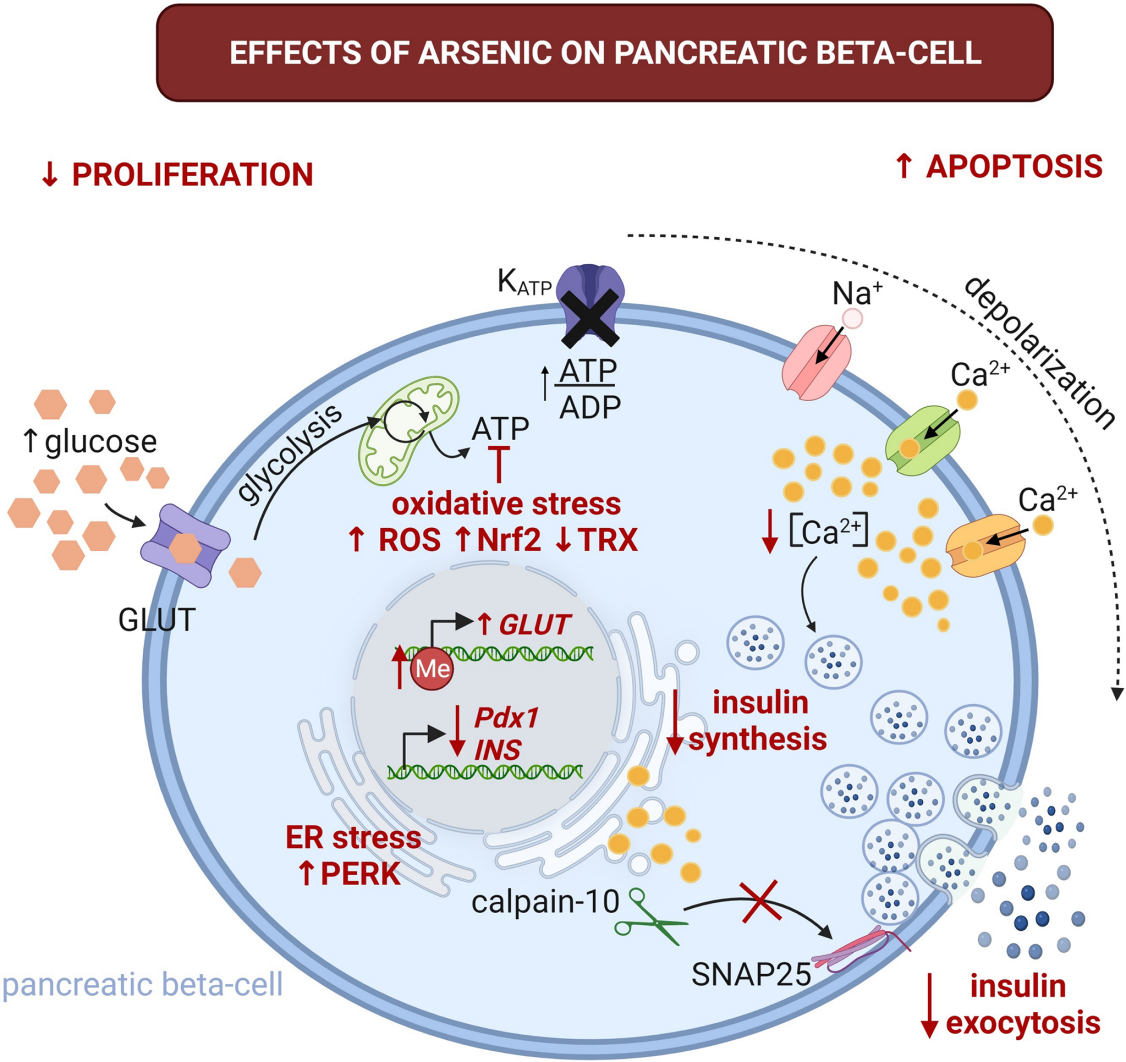
Effects of arsenic on pancreatic beta-cell physiology. The alterations induced by this pollutant are highlighted with red font. Arsenic could promote glucose intolerance and T2D through beta-cell damage and impaired GSIS. It promotes beta cell apoptosis, while inhibiting proliferation. This can lead to imbalance in beta-cell mass contributing to decreased insulin secretion and promoting the development of T2D. Arsenic severely impairs glucose tolerance by the reduction of GSIS. It affects insulin synthesis by decreasing insulin mRNA (INS) levels, increasing endoplasmic reticulum (ER) stress and activation of protein kinase RNA-like endoplasmic reticulum kinase (PERK). Arsenic can also impair GSIS through several mechanisms, including oxidative stress by altering mitochondrial metabolism, this can also lead to alterations in the ATP/ADP ratio, which is an essential step for GSIS. Arsenic inhibits the oscillations of intracellular Ca^2+^ and reduces the proteolysis of synaptosomal-associated protein 25kDa (SNAP-25) by calpain-10 protease. These alterations result in decreased insulin secretion. GLUT, glucose transporter (GLUT1 and 3 in humans, GLUT2 in rodents); Me, 5-methylcytidine; Nrf2, nuclear factor erythroid 2-related factor 2; Pdx1, pancreatic and duodenal homeobox 1; ROS, reactive oxygen species; TRX, thioredoxin. Created with BioRender.com.

Although epidemiological studies have demonstrated a diabetogenic effect of arsenic, the mechanisms remain largely unknown. Nevertheless, evidence indicates that dysfunction of pancreatic beta-cells is one of the significant mechanisms of arsenic-induced T2D (**Figure 1**) (46–48).

Glucose-stimulated insulin secretion (GSIS) starts when glucose enters the beta-cells through glucose transporters (GLUT1 and GLUT3 in humans, and GLUT2 in rodents) (49). Then, glucose is metabolized through the glycolytic pathway and Kreb’s cycle, increasing ATP synthesis (50). The rise in intracellular ATP/ADP ratio leads to the closure of the ATP-sensitive potassium channels, depolarizing the membrane potential, which opens sodium and calcium channels, inducing action potentials, leading to the increase in intracellular calcium and insulin exocytosis (50, 51).

Sodium arsenite affects the GSIS in a dose- and time-dependent fashion (52). In male Wistar rats treated with sodium arsenite at 1.7 mg/kg (by gavage every 12 h for 90 days) causes hyperglycemia, hyperinsulinemia and insulin resistance (46). Interestingly, similar effects were observed in male Sprague-Dawley rats treated with sodium arsenite doses of 2.5 and 5 mg/kg (via oral gavage for three months). At the end of treatment, the animals develop hyperinsulinemia, increased HOMA-IR, glucose intolerance and insulin resistance in a dose-dependent fashion (53). Both models are relevant because resemble the MS condition, suggesting that arsenic could be involved in the pathophysiology of MS (**Table 1**). In contrast, C57BL/6J male mice treated with 50 ppm of sodium arsenite for 8 weeks exhibited impaired glucose tolerance compared to controls, without inducing peripheral insulin resistance. Moreover, beta cells from treated mice showed decreased insulin secretion without changes on pancreatic beta-cells mass (61). Thus, suggesting that arsenic disrupts beta cell function independently of its cytotoxicity. In four-week-old male weanling C57BL/6J mice treated with 25 or 50 ppm of sodium arsenite and high-fat-diet (HFD) for 20 weeks resulted in an increased glucose intolerance and decreased insulin secretion in dose-dependent manner compared with mice only exposed to HFD (32).

**Table 1:** Comparison of arsenic exposure effects on the signs of MS between epidemiological studies and animal models.

Model (species)	Arsenic dose (time)	MS-related effects	Ref.
**Epidemiological studies**			
Taiwanese population	Arsenic in water in non-MS patients: 569.94 ± 321.51 ppb Arsenic in water in MS-patients: 684.39 ± 245.93 ppb	↑ MS (DMA%)	(14)
American Indian communities	Arsenic metabolites assessed in urine Total urinary arsenic 6.5 ppb	↑ MS (DMA%) ↑ BMI (DMA%) ↑ Fasting glucose = HDL (DMA%) = TAG (DMA%) = Hypertension (DMA%) = HOMA-IR	(15)
Iranian population	Arsenic in water 257 to 342 ppb. Urinary arsenic was measured in subjects Total urinary arsenic 3 ppb	↑ MS (DMA% and DMA/MMA)	(16)
Chilean population	Cumulative arsenic exposure lagged 40 years: 610–5279 ppb	↑ Diabetes in obese individuals	(24)
Adult women U.S. population	Urinary total arsenic and speciated arsenic Total urinary arsenic 6.5 ppb	High BMI associated with lower arsenic methylation	(25)
Adult women Chilean population	Urinary total arsenic adjusted for creatinine Total urinary arsenic 14 ppb	= BMI = Fat mass percentage	(26)
Bangladeshi population	Cumulative arsenic exposure lagged 10 years: >50–250 ppb	↑ Diabetes in exposures higher than 50 ppb = BMI	(27)
Korean population	Total urinary arsenic ranging from 0.36-36.7 ppb	= BMI ↓ HDL	(28)
Adult Welders US Population	Arsenic in toenails 0.18 (0.15) mg/g toenail.	Inverse correlation between BMI and toenail arsenic	(29)
Non-Hispanic white, Non-Hispanic black, Mexican American and Other Hispanic populations	Urinary arsenic	Inverse correlation between excreted arsenic and BMI	(30)
Taiwanese population	Arsenic concentrations in water 700 to 930 ppb)	↑ T2D = BMI	(47)
Bangladeshi population	142 ± 278 ppb water in non-T2D 202 ± 277 ppb in the water of T2D	↑ T2D	(54)
Taiwanese children and adolescents	Urinary total arsenic and speciated arsenic Total urinary arsenic 27.06 ppb	↑ HOMA-IR (higher effect of arsenic in subjects with high BMI)	(55)
Bangladeshi population	Mean arsenic concentration in water of 129.5 ppb	= BMI ↑ Insulin resistance HOMA-IR ↑ Fasting blood glucose ↓ Lean body mass ↓ HOMA-B in females ↑ Hyperinsulinemia	(56)
US adolescents	Urinary total arsenic and speciated arsenic Total urinary arsenic 3.77 ppb	= HOMA-IR	(57)
Mexican population	Arsenic in water: 77.3 ppb in T2D 39.2 ppb in non-T2D	↑ HbA1c ↓ Hyperinsulinemia ↓ HOMA-IR	(58)
Bangladeshi population	Arsenic in drinking water 173.46 ppb	↓ Cholesterol, LDL and HDL ↑ Oxidized LDL	(59)
Chinese population	Arsenic measured in plasma (no concentrations available)	↑ Dyslipidemia ↓ HDL	(60)
**Mouse models**			
Adult C57BL/6 male mice	Sodium arsenite 25 and 50 ppm (8 weeks)	↑ Glucose intolerance (50 ppm) = Body weight	(31)
Adult C57BL/6 male mice	Sodium arsenite 25 and 50 ppm (20 weeks) + HFD	↓ Weight gain and adiposity (25 and 50 ppm + HFD) ↑ Liver TAG (25 ppm + HFD) ↑ Glucose intolerance (25 and 50 ppm + HFD) ↓HOMA-IR (25 and 50 ppm+ HFD)	(32)
Adult C57BL/6J male mice	Sodium arsenite 50 ppm (16 weeks) + HFD	= Diet-induced body weight gain ↓ Diet-induced visceral adiposity ↓ Diet-induced liver TAG ↓ HOMA-IR ↓ HOMA-β	(33)
Swiss Webster male mice *in utero* (IU) or *in utero* until adulthood (IU+) or adulthood only (PN)	Sodium arsenite 100 ppb (from *in utero* until post-natal week 13)	↑ TAG in IU and IU+ ↑ Cholesterol in IU+ ↑ FFA in IU+ ↑ NAFLD in IU and IU+ ↑ Body weight in IU+ ↑ HOMA-IR in IU+	(37)
Female offspring from CD-1 mice treated *in utero*	Sodium arsenite 10 ppb and 42.5 ppm *In utero*	↑ Body weight and adiposity (both doses) ↑ Glucose intolerance ↑ Hyperinsulinemia	(38)
Adult C57BL/6J male mice	Sodium arsenite 50 ppm (8 weeks)	= Body weight ↑ Glucose intolerance = Insulin resistance (ITT) ↓ HOMA-IR	(61)
Adult female and ovariectomized ICR mice	0.05 and 0.5 ppm (6 weeks)	↑ HOMA-IR in ovariectomized females + 0.05 and 0.5 ppm arsenic ↑ Fasting glucose ↓ Fasting insulin in sham females + arsenic ↑ Hyperinsulinemia in ovariectomized females + arsenic ↑ Insulin resistance in ovariectomized females + arsenic (ITT) ↑ Glucose intolerance in ovariectomized females + arsenic	(62)
Adult NMRI male mice	Sodium arsenite 25 and 50 ppm (20 weeks) + HFD	↓ Diet-induced body weight ↓ Diet-induced fasting glucose ↑ Glucose intolerance ↓ Diet-induced HOMA-IR ↓ Diet-induced insulin resistance (ITT) ↓ Diet-induced dyslipidemia	(63)
Adult non-diabetic male C57BLKS/J db/m mice and diabetic C57BKS/Leprdb (db/db) mice	Sodium arsenite 3 ppm (16 weeks)	= Body weight gain in both mice models ↑ Fasting glucose in db/db mice + arsenic ↑ Hyperinsulinemia in db/m mice + arsenic ↓ Insulin levels in db/db mice + arsenic = HOMA-IR in both mice models ↑ Glucose intolerance in db/db mice + arsenic ↓ Insulin resistance in db/db mice + arsenic (ITT) ↑ FFA in adipose tissue	(64)
**Rat models**			
Adult male Wistar rats	Sodium arsenite 3.4 mg/kg/day (90 days)	↑ Fasting glucose ↑ Hyperinsulinemia ↑ HOMA-IR	(46)
Adult male Sprague-Dawley rats	Sodium arsenite 2.5 and 5 mg/kg/day (3 months)	↑ Hyperinsulinemia ↑ HOMA-IR ↑ Glucose intolerance ↑ Insulin resistance (ITT) ↑ Liver TAG ↑ Non-alcoholic steatosis	(53)
Adult male Sprague-Dawley rats	Sodium arsenite 2.5 and 5 mg/kg/day (5 months)	↓ Fasting insulin	(65)
Adult Sprague-Dawley rats	Polluted water containing 53 ppb arsenic and 30 ppb of lead (3 months)	↑ Glucose intolerance in females and males ↑ Hyperinsulinemia during OGTT in males ↑ HOMA-IR in males ↑ TAG in females ↑ Cholesterol and LDL in males	(66)
Adult male Wistar rats	Sodium arsenite 50 ppm, 100 ppm and 150 ppm Sodium arsenate 100 ppm, 150 ppm and 200 ppm (12 weeks)	↓ Cholesterol and HDL by arsenite ↑ Cholesterol by arsenate ↓ HDL by arsenate ↑ TAG (50 ppm arsenite and 100, 150 and 200 ppm arsenate) ↑ FFA by both arsenicals ↑ TAG in liver (arsenite 50 ppm and arsenate 150 and 200 ppm) ↓TAG in liver (arsenite 100 and 150 ppm)	(67)
Adult male Wistar rats	Sodium arsenite 1.5 mg/kg/day (28 days)	= Weight gain ↑ Cholesterol and LDL ↓ HDL	(68)
Adult male Wistar rats	Sodium arsenite 5 mg/kg/day (4 weeks)	↑ Cholesterol ↑ TAG, FFA, LDL ↓ HDL ↓ Weight ↑ Heart inflammation and ROS	(69)
Adult male Wistar rats	Sodium arsenite 50 ppm Sodium arsenate 50 ppm (200 days)	↓ Weigh (arsenite) ↑ Hypertension (both arsenicals) ↑ TAG and cholesterol (both arsenicals)	(70)

Note that while humans are exposed to arsenic doses in the range of ppb (10^-6^ g/L), most animal studies are performed with doses in the range of ppm (10^-3^ g/L).

DMA dimethylated arsenic, FFA free fatty acids, HbA1c glycated hemoglobin, HDL high density lipoprotein, HFD high fat diet, ITT insulin tolerance test, LDL low density lipoprotein, MS metabolic syndrome, NAFLD non-alcoholic fatty liver disease, OGTT oral glucose tolerance test, ROS reactive oxygen species, T2D type 2 diabetes, TAG triglycerides. ↑ arsenic induced an increase in the indicated parameter; ↓ arsenic induced a decrease in the indicated parameter; = arsenic did not induce an effect.

Pancreatic beta-cells isolated from male Wistar rats treated *in vitro* with up to 5 µM of sodium arsenite for 72 and 144 h resulted in a significant reduction of GSIS (71). Moreover, insulin mRNA levels decreased after 72 h of treatment with 5 µM of sodium arsenite (71). Concordantly, in the insulinoma cell line INS-1(832/13), treatment with up to 5 µM of sodium arsenite for 96 h resulted in a dose-dependent reduction of GSIS. However, insulin secretion triggered by KCl increased to 5 µM of sodium arsenite. In addition, in this cell line, the insulin gene expression was increased by sodium arsenite in a dose-dependent fashion (72). Other studies showed that the treatment with concentrations of sodium arsenite up to 2 µM in pancreatic islets from C57BL/6 mice, or RINm5F insulinoma cells attenuate GSIS without affecting insulin synthesis (20, 73). Thus, indicating that arsenic can affect insulin synthesis and secretion.

GSIS is a complex mechanism that involves biochemical and electrical events (see *The Effects of Arsenic on Ionic Channel Function as a Mechanism for Arsenic-Induced Metabolic Alterations*). On the other hand, the *in vivo* and *in vitro* models to study this mechanism are diverse, so it is difficult to draw a full picture of the progression of the events toward pancreatic beta-cell dysfunction. We consider that each model’s susceptibility to arsenic toxicity in could explain some of the observed discrepancies. As mentioned before, arsenic metabolism is different between rats and mice. In rats, the hemoglobin in erythrocytes sequestrates arsenic, and there are differences in the ratios of DMA/MMA/inorganic arsenic excreted in urine (11, 18).

Several mechanisms for the effects of arsenic on GSIS are described. During insulin exocytosis, it is possible that the calpain-10 protease is activated in response to Ca^2+^ influx, partially proteolyzing the synaptosome-associated protein 25 (SNAP25), triggering the exocytosis of insulin (74). A decrease in the oscillations of intracellular Ca^2+^, reducing the proteolysis of SNAP-25, and impairing insulin secretion was observed in RINm5F insulinoma cells treated with 1 µM of sodium arsenite for 72 h. Furthermore, sodium arsenite treatment reduced proliferation in this beta cell line (73).

Other mechanisms indicate different roles for oxidative stress and reactive oxygen species (ROS) induced by arsenic. This observation is particularly relevant because the pancreas has low antioxidant capabilities and is susceptible to arsenic-induced oxidative stress (44). INS-1 832/13 insulinoma cells treated with sodium arsenite (2.5 -10 µM) showed increased intracellular ROS levels and apoptosis (54). Sodium arsenite treatment (2 µM) for 24 h inhibited oxygen consumption rate (OCR) in both glucose- and pyruvate-stimulated INS-1 832/13 cells, reducing GSIS (75), suggesting alterations in mitochondrial metabolism that could result in an imbalance of the ATP/ADP ratio which is an essential factor for GSIS. In addition, a study carried out in rats subjected to sodium arsenite doses of 2.5 and 5 mg/Kg/day (by oral gavage) for 5 months, showed that ferroptosis (a form of cell death triggered by lipid peroxidation in an iron-dependent way) is involved in arsenic-induced pancreatic b cells damage. This ferroptosis is regulated by ferritin through the MtROS-autophagy (65).

ROS derived from glucose metabolism is a metabolic signal for GSIS, and Nrf2 is a central transcription factor that regulates the adaptive cellular response to oxidative stress (76, 77). Treatment with 0.25 and 0.5 µM of sodium arsenite for 96 h to INS-1 832/13 cells led to an increase of the antioxidant response of Nrf2 activity, which was associated with decreased GSIS (72). Thioredoxin reductase (TRX) is another enzyme that protects cells from oxidative damage (78). In INS-1 832/13 cells, the exposure to 0.25-1 µM of sodium arsenite for 96 h decreased cell viability and TRX activity in a dose-dependent manner (79). Concordantly, the enzymatic activity of TRX in pancreatic tissue was lower than the control group in an *in vivo* study with male Wistar rats, treated with sodium arsenite at 1.7 mg/kg orally for 90 days (46). These works indicate that oxidative damage in beta cells, along with altered antioxidant signaling, can lead to impaired GSIS.

Endoplasmic reticulum (ER) stress is an additional mechanism involved in impaired GSIS induced by arsenic. Sodium arsenite (4 µM) treatment for up to 24 h impaired GSIS and induced ER stress by activating the eukaryotic translation initiation factor 2 alpha kinase 3 (PERK) in INS-1 cells. GSIS was restored by adding a PERK inhibitor to the sodium arsenite-treated cells, demonstrating the relevance of this mechanism (80).

Environmental pollutants frequently exert their toxic effect through epigenetic modifications. Pancreatic islets from male Wistar rats treated with 1 µM of sodium arsenite for 6 days showed impaired GSIS. In these islets, sodium arsenite increased *Glut2* gene expression due to changes in the DNA methylation of the *Glut2* gene promoter. In contrast, the expression of insulin and Pdx1 was significantly reduced without changes in the methylation pattern of these genes (81). Although human beta-cells do not depend on GLUT2 for glucose uptake and GSIS (49), the genes encoding GLUT1 and GLUT3 transporters could have altered methylation patterns because arsenic can up- and down-regulate DNA methylation in a *loci*-dependent manner (82). However, this possibility should be directly tested in human islets exposed to arsenic.

Another mechanism for the arsenic effects on GSIS involves serotonin metabolism. Exposing MIN6-k8 mouse beta-cell line to arsenite (0.1-1 µM) for three days decreased GSIS and reduced serotonin and its precursor 5-hydroxytryptophan (5-HTP). In addition, arsenic increased mRNA levels of the polypeptide a6a enzyme gene (Ugt1a6a), which promotes the disposal of cyclic amines *via* glucuronidation. Interestingly, this effect was prevented by knockdown of Ugt1a6a during arsenite exposure in MIN6-K8 cells, restoring GSIS (83).

Whether this array of mechanisms in the beta cells coincide or is induced under specific arsenic concentrations or exposure time remains to be explored. However, the present evidence supports the notion that deregulation of beta cell physiology and GSIS is one of the primary mechanisms for arsenic-induced glucose intolerance.

## Mechanisms for Arsenic-Induced Insulin Resistance

Insulin orchestrates anabolic processes in its metabolic target tissues during postprandial state, mainly the liver, skeletal muscle, and adipose tissues (84). The binding of insulin to the insulin receptor (IR) in the target cells results in the transphosphorylation of tyrosine residues, increasing its kinase activity. Among the signal transduction pathways activated by insulin, the canonical way involves the activation of the phosphatidylinositol 3 kinase (PI3K) and the Akt protein kinase (85). This pathway regulates different downstream proteins that mediate many of the processes regulated by insulin (84, 86). Insulin activates glycogen synthesis and lipogenic pathways in the liver while inhibiting glycogenolysis and gluconeogenesis. In the skeletal muscle, insulin induces glucose uptake and glycogen synthesis. In the adipose tissue, insulin inhibits lipolytic pathways and induces glucose uptake and lipogenesis (84, 87, 88). Thus, insulin resistance is a pathophysiological condition characterized by a reduced insulin response by peripherical tissues, contributing to hyperglycemia, dyslipidemia, hyperinsulinemia and other alterations (2, 89).

Because of the wide range of insulin actions in the body, it is anticipated that assessing insulin resistance can be challenging. This can be reflected by the fact that in some epidemiological studies, arsenic exposure is linked to insulin resistance (55, 56, 90), but not in others (15, 57, 58). Nevertheless, most epidemiological studies assess insulin resistance by calculation the homeostatic model assessment for insulin resistance (HOMA-IR), while there are no studies evaluating insulin resistance by the golden standard, the hyperinsulinemic-euglycemic clamp (89). Although this approach results more invasive to the patients, it allows to effectively measure whole body insulin resistance. Future studies using this approach will help to elucidate whether arsenic is associated to insulin resistance in exposed populations.

Concordantly with epidemiological evidence, in animal models, arsenic treatment is not always related to insulin resistance (**Table 1**). In female outbred ICR mice exposed to 0.25 and 0.5 ppm of sodium arsenite for 6 weeks did not develop insulin resistance. However, ovariectomized females developed insulin resistance in a dose-dependent fashion, which was prevented by estradiol supplementation (62). Implying that arsenic exposure could interact with estrogen signaling to determine whether the organism develops insulin resistance or not. In Sprague-Dawley rats consuming water with 53 ppb of arsenic and 30 ppb of lead for 3 months, only the males developed insulin resistance, while both males and females developed glucose intolerance (66). Likewise, male Sprague-Dawley rats receiving 2.5 and 5 mg/kg/day of arsenite for three months developed insulin resistance (53). In contrast, male C57BL/6J mice exposed to 50 ppm of sodium arsenite for 8 weeks developed glucose intolerance without impairment in insulin sensitivity (61). These works indicate that insulin resistance induced by arsenic could be dependent on other factors, such as sexual dimorphism and arsenic concentration. In this regard, it is known that arsenic interacts directly with the carbon structure of 17-beta-estradiol (91). Also, arsenic modulates androgen, progesterone, estrogen, glucocorticoid and mineralocorticoid steroid hormones-regulated genes in a non-monotonic fashion (92). These data imply that the interaction between sex and arsenic exposure is complex, so studies using both sexes and a wide range of arsenic concentrations will help elucidate in which cases arsenic can contribute to the development of insulin resistance.

The possible interactions between arsenic consumption, diet, genetic factors, and the development of insulin resistance in animal models have been tested. In outbred NMRI male mice, consumption of 25 and 50 ppm of sodium arsenite for 20 weeks reduced the insulin resistance induced by high-fat diet intake. Nevertheless, the simultaneous exposure to arsenic and a high-fat diet resulted in higher glucose intolerance (63). Similarly, non-diabetic male C57BLKS/J db/m mice treated with 3 ppm of sodium arsenite for 16 weeks did not develop insulin resistance. Still, in diabetic C57BKS/Leprdb (db/db) mice, the same treatment improved insulin sensitivity, compared with non-treated diabetic mice (64). Interestingly, C57BL/6J mice of both sexes developed insulin resistance by 100 ppb of sodium arsenite for 24 weeks only when fed with a high-fat, low-folate diet. Although folate is an important nutrient necessary for arsenic metabolism and detoxifying mechanisms (93), the effect of low-folate diet on insulin resistance induced by arsenic was independent of the arsenic metabolism (94). Folate is an essential vitamin that participates in enzymatic reactions involved in the one-carbon metabolic pathways, regulating DNA methylation, and amino acid and lipid metabolism (95). Interestingly, epidemiological evidence indicates that diet supplementation with folate improves glycemic control and insulin resistance in patients with MS and T2D (95, 96). These lines of evidence let us suggest that arsenic and folate can have antagonistic effects on the same metabolic pathways, influencing the development of insulin signaling. Nevertheless, this hypothesis requires experimental validation in future studies.

As mentioned above, exposure to endocrine-disrupting chemicals during critical development windows can promote physiological and metabolic alterations that persist until adulthood (97). Similar to the effects of intrauterine arsenic exposure on obesity, the male offspring from C57BL/6J mice dams treated with 100 and 1000 ppb of sodium arsenite during pregnancy developed insulin resistance. However, this effect was not observed in female offspring (98). In contrast, female offspring from sire C57BL/6J mice treated with 250 ppb of sodium arsenite for 3 weeks developed hepatic insulin resistance. However, this effect was absent in the male offspring (99). Thus, indicating that exposure to arsenic during intrauterine development can increase the risk of developing insulin resistance during adulthood. Altogether, the evidence shows that the interaction between arsenic exposure and insulin signaling is not necessarily linear, and factors such as sex, development window, nutrient intake and genetic background will influence the development of insulin resistance. Another possible explanation for the discrepancy between the studies is the method used to assess insulin resistance. While some works only reported the HOMA-IR (66, 94, 98), some studies performed the insulin-tolerance test (ITT) (53, 61–64, 99). Likewise, studies performing ITT used totally different insulin doses, ranging from 0.75 mIU/kg to 1.5 IU/kg. Future studies need to use more homogeneous methods to assess insulin resistance in animal models. Also, there is an urgent need to evaluate the development of this condition by the golden standard the hyperinsulinemic-euglycemic clamp. These two approaches will allow to confirm under which conditions arsenic interferes with insulin signaling and will make easier to compare the findings between studies. A second possibility is that arsenic induces tissue- and pathway-specific alterations in insulin signaling, that do not necessarily result in alterations in whole-body glycemia. For instance, insulin resistance can result in altered lipogenesis in the liver and adipose tissue, increased lipolysis in the adipose tissue, altered amino acid metabolism in the muscle and hyperinsulinemia. These possibilities deserve further clarification in future studies.

**Figure 2:**
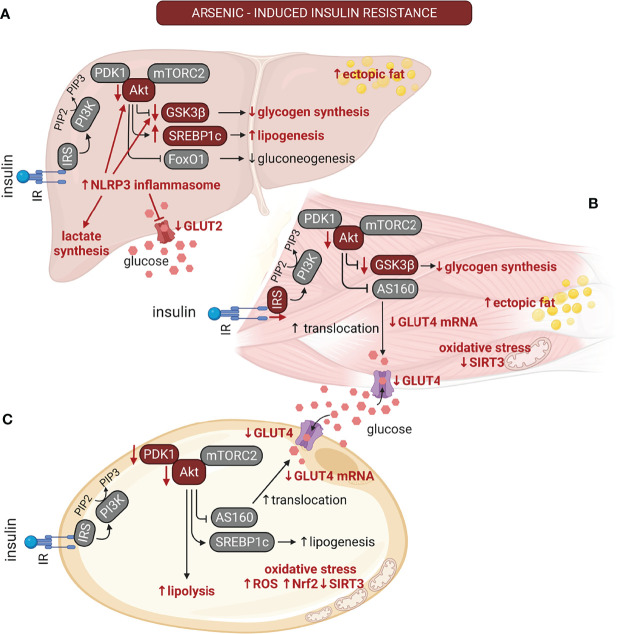
The multiple effects of arsenic on insulin signaling are tissue-dependent. The alterations induced by arsenic exposure on the insulin signaling pathway are depicted with red font. Arsenic induces insulin resistance in classical insulin targeted organs. **(A)** In liver, arsenic reduces GLUT2 protein levels and reduces glycogen synthesis due to NLR family pyrin domain containing 3 (NLRP3) inflammasome activation. This also results in reduction in the phosphorylation of Akt and glycogen synthase kinase 3β (GSK3β). Also, it promotes liver lipogenesis and the development of non-alcoholic fatty liver disease (NAFLD). **(B)** In muscle, arsenic inhibits GLUT4 expression. It also reduces insulin signaling by inhibiting IRS-1 and Akt phosphorylation, reducing insulin-stimulated GLUT4 translocation and decreased glycogen levels, thus contributing to hyperglycemia in the postprandial state. Arsenic can also promote ectopic fat deposition in muscle. **(C)** In adipose tissue, this pollutant induces adipocyte hypertrophy, impaired adipogenesis, and suppression of mitochondrial biogenesis. Arsenic stimulates lipolysis, and oxidative stress due to reduced SIRT3 activity. On the other hand, it inhibits GLUT4 expression and insulin-stimulated GLUT4 translocation by inhibition of Akt phosphorylation. Akt, protein kinase B; AS160, Akt substrate 160 kDa; FoxO1, forkhead-box-O1; GLUT4, glucose transporter 4; GSK3β, glycogen synthase kinase 3β; IR, insulin receptor; IRS, insulin receptor substrate; mTORC2, mammalian target of rapamycin complex 2; NLRP3, NLR family pyrin domain containing 3; Nrf2, nuclear factor erythroid 2-related factor 2; PDK1, Phosphoinositide-dependent kinase-1; PI3K, phosphatidyl inositol 3 kinase; PIP2, phosphatidyl inositol 4;5-biphosphate; PIP3, phosphatidyl inositol 3;4;5 triphosphate; ROS, reactive oxygen species; SIRT3, sirtuin-3; SREBP1c, sterol regulatory element-binding transcription factor 1c. Created with BioRender.com.

Several mechanisms are described for arsenic-induced insulin resistance (**Figure 2**). One of the main effects of insulin on the adipose tissue and the skeletal muscle is to increase glucose uptake, which is mediated by the increase in vesicular trafficking of the glucose transporter-4 (GLUT4) from intracellular compartments to the plasma membrane, resulting in insulin-stimulated glucose uptake (ISGU) (87). In insulin-resistant individuals, the defects in this process contribute to hyperglycemia in the postprandial state (100). In 3T3-L1 adipocytes, treatment with sodium arsenite (up to 100 µM for 3 h), and its methylated metabolites inhibited ISGU in a concentration- and time-dependent fashion. This effect was due to reduced levels of Akt and 3 phosphoinositide-dependent kinase-1 (PDK1) (21, 101).

Redox balance and ROS production are important mediators of insulin signaling (102). Treating 3T3-L1 adipocytes with lower sodium arsenite concentrations (up to 2 µM for 7 days) resulted in the activation of the Nrf2-dependent antioxidant pathway. This pathway deregulated insulin-induced ROS production, impairing Akt phosphorylation and ISGU. Moreover, sodium arsenite treatment induced the expression of inflammatory response genes, which could contribute to insulin resistance (103). Similarly, a study carried out in 3T3-L1 preadipocytes and C2C12 myoblasts treated with sodium arsenite up to 2 µM for 8 weeks prior inducing their differentiation into mature adipocytes and myotubes, respectively, showed that sodium arsenite reduced ISGU by decreasing GLUT4 expression, while induced oxidative stress due to reduced Sirtuin-3-dependent mitochondrial antioxidant pathways (104). Nevertheless, the experimental design of this work do not allow to point out whether the effect of arsenic on ISGU was due to alterations in insulin signaling, or a defect in the differentiation of these cell lines. In this regard, it is known that the GLUT4 storage vesicles (GSV), which are the main source of GLUT4 after insulin stimulation, are formed exclusively in matured adipocytes and muscle fibers (105). Highlighting the need to dissect between defects in insulin signaling, or impairment in cell differentiation. In the skeletal muscle of C57BL/6J mice, the treatment with 4 ppm of arsenic trioxide for 12 weeks reduced muscle glycogen (106). Intriguingly, arsenic trioxide induced the inhibitory phosphorylation of glycogen synthase kinase-3 (GSK3), indicating that the reduction in glycogen levels could be due to other alterations in the glycogen synthesis pathway.

Moreover, arsenic treatment inhibited IRS1 and Akt phosphorylation and induced ectopic fat accumulation in the muscle fibers (106), which are markers of insulin resistance in muscle (100). In addition, increased autophagy in muscle cells accompanied alterations in the insulin signaling pathway (100, 106). Largely, the data about the effects of arsenic on the insulin signaling suggest that Akt is a primary target for impaired ISGU. Nevertheless, is important to note that it is necessary to block more than 90% of the Akt activity in muscle *in vivo* to effectively impair GLUT4 translocation and ISGU (107, 108). Thus, it is hypothezysed that insulin resistance could also be determined by sorting of GLUT4 into different vesicle compartments, or with alterations in distal sections of insulin signaling (87, 108). It will be interesting to test whether arsenic can affect these pathways to impair ISGU in different muscle groups and fat depots.

The main actions of insulin in the liver are inhibiting hepatic glucose release by suppressing glycogenolysis and gluconeogenesis while promoting glycogen synthesis, lipogenesis and lipid export to other tissues (109). Primary murine hepatocytes treated *in vitro* with sodium arsenite (up to 2 µM) or methylarsine oxide (up to 1 µM, a monomethylated metabolite of arsenic) for 4 hours had a reduction in insulin-stimulated Akt phosphorylation, which is a critical node in insulin signaling that regulates glycogen metabolism (110). Concordantly, sodium arsenite reduced glycogen synthesis due to lower activity of the glycogen synthase (GS) and higher activity of the glycogen phosphorylase (GP), which regulate the synthesis and degradation of glycogen, respectively (110). Co-treatment with sodium arsenite and lipopolysaccharides (LPS) in L-02 hepatocarcinoma cell line induced NLRP3 inflammasome activation, leading to reduction in Akt and glycogen-synthase kinase 3β (GSK3β) phosphorylation, and increased glycogen synthase phosphorylation levels (111). A similar study found that NLRP3 inflammasome inhibitors prevented arsenic and LPS-induced insulin resistance in HepG2 hepatocarcinoma cells (53). Interestingly, NLRP3 inflammasome activation in the liver of rats treated with 2.5 and 5 mg/kg/day of sodium arsenite for three months resulted in diminished liver glycogen levels and increased triglyceride levels (53). NLRP3 induction by sodium arsenite exposure *in vivo* reduced GLUT2, glucokinase, and pyruvate kinase protein abundance, while increasing lactate dehydrogenase levels in the liver of the treated rats. Thus, switching the glycolytic flux from the oxidative pathway to lactate synthesis (111). In mice treated with sodium arsenite (up to 4 mg/L) for 12 weeks, the liver had higher levels of triglycerides and overexpression of gluconeogenic enzymes (112). Also, the liver of these mice exhibited reduced GLUT2 protein levels and lower phosphorylation of Akt and GS, which could be related to the diminished glycogen levels observed (112). Arsenic tretament in normal C57BLKS/J db/m and diabetic C57BKS/Leprdb (db/db) mice (3 ppm for 16 weeks) resulted in increased free fatty acid accumulation in liver and gluconeogenesis (64). Therefore, arsenic causes liver insulin resistance both *in vitro* and *in vivo*, resulting in lower glycogen levels, increased liver lipids and gluconeogenesis. Although the accumulation of lipids in the liver of treated animals could indicate the development of liver steatosis or NAFLD, further work focused on these diseases are needed to elucidate all the morphophysiological alterations that occur during the development of these diseases.

In addition to the cell-dependent effects of arsenic to induce insulin resistance, another possibility can be expected by the fact that arsenic compounds can bind proteins through different chemical and electrostatic bonds, altering protein function. Specifically, trivalent arsenicals bind to the sulfhydryl groups of proteins, depending on the surrounding amino acid chains (17). One study showed that arsenite could bind insulin directly, changing its stability and lowering its melting temperature, which could be reversed by encapsulating insulin in poly (lactic-co-glycolic) acid nanoparticles (113). Importantly, the authors also showed that this encapsulated insulin has a higher impact on controlling glycemia than insulin alone in Swiss mice of both sexes treated with arsenite (20 mg/kg) for 8 weeks (113).

## Effects of Arsenic Exposure on Lipid Metabolism

**Figure 3:**
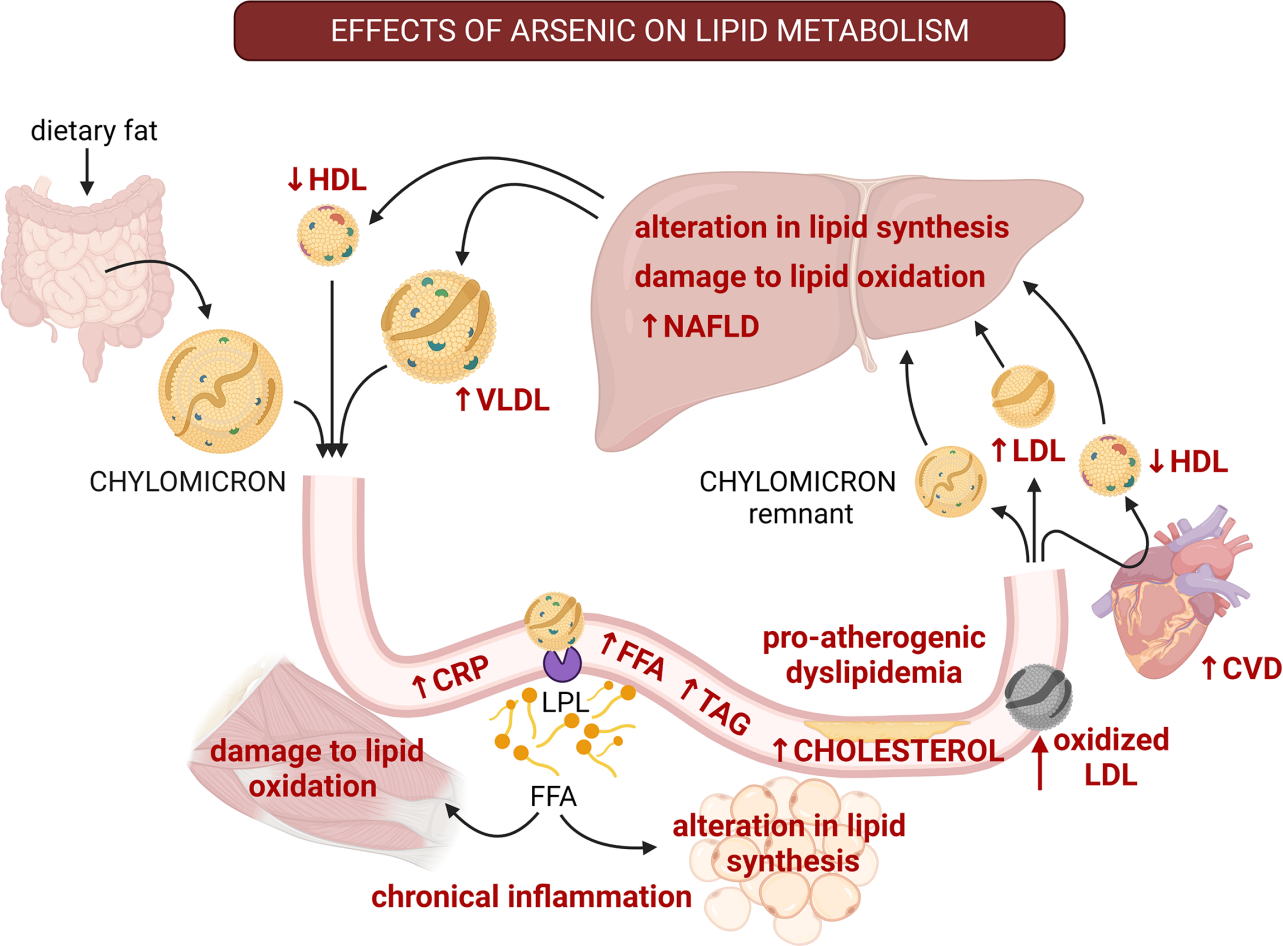
The multiple effects of arsenic on lipid homeostasis and the progress to pro-atherogenic dyslipidemia. The effects of arsenic are shown with red font. Arsenic alters synthesis and oxidation of lipids in liver, promoting the development of non-alcoholic fatty liver disease (NAFLD). Also, arsenic diminishes HDL-cholesterol, and increases LDL-cholesterol, free fatty acids, and triglycerides, which are characteristics of pro-atherogenic dyslipidemia. Besides, arsenic promotes lipid peroxidation, which also promotes atherosclerosis. Arsenic exposure correlates with high levels of oxidized LDL and C-reactive protein (CRP) which contributes to systemic inflammation and alterations to lipid metabolism in other tissues. These alterations lead to insulin resistance and are a major risk factor for developing cardiovascular diseases. CVD, cardiovascular disease; CRP, C-reactive protein; FFA, free fatty acids; HDL, high-density lipoprotein; LDL, low-density lipoprotein; LPL, lipoprotein lipase; TAG, triglycerides; VLDL, very low-density lipoprotein. Created with BioRender.com.

MS may present several alterations in lipid homeostasis, including hypertriglyceridemia, high plasma levels of low-density lipoproteins (LDL) and free fatty acids (FFA), and low plasma levels of high-density lipoproteins (HDL) (114). These alterations lead to insulin resistance, chronic inflammation, NAFLD and are a major risk factor for developing CVD (115). In human populations, arsenic exposure correlated with low levels of HDL, high levels of LDL, oxidized LDL, and C-reactive protein (CRP) (**Figure 3**) (59, 60). Also, a recent meta-analysis found that arsenic exposure through drinking water is associated with low levels of HDL and high levels of LDL, while it was not associated with plasmatic triglyceride and total cholesterol levels (116). Low levels of HDL along with high levels of oxidized LDL and chronic inflammation are associated with coronary artery disease, atherogenic lesions, and impaired metabolic health in patients with obesity and MS (117–119). Thus, arsenic exposure could promote the development of pro-atherogenic dyslipidemia.

In male Wistar rats treated with sodium arsenite (up to 150 ppm) or arsenate (up to 200 ppm) for twelve weeks, a differential pattern of dyslipidemia was induced by each arsenic species (67). While sodium arsenite-induced hypocholesterolemia and accumulation of cholesterol in the liver, arsenate produced hypercholesterolemia and hypertriglyceridemia (67). Moreover, both arsenicals reduced HDL concentrations in plasma, and promoted high levels of free fatty acids (67). Another study on male adult Wistar rats administered with sodium arsenite (1.5 mg/kg/day by gavage for 28 days) found low levels of HDL and high levels of LDL (68). Interestingly, this effect was prevented by the administration of gadolinium chloride, which is an inhibitor of liver-residing macrophages known as Kupffer cells (68). Similarly, 5 mg/kg/day of sodium arsenite for four weeks resulted in dyslipidemia; characterized by hypercholesterolemia, hypertriglyceridemia, high levels of serum phospholipids, free fatty acids, LDL and very-low-density lipoproteins (VLDL), and low levels of HDL in male Wistar rats (69). Moreover, these alterations caused cardiotoxicity (69). All the toxic effects induced by arsenic were prevented by ingestion of silibinin, a liver-protecting compound (69). These works point to the notion that liver toxicity induced by arsenic drive the development of dyslipidemia even in the absence of hypercaloric diets. Since the liver is the main organ responsible for arsenic metabolism, and it is exposed to higher arsenic concentrations through the portal circulation which transports the arsenic ingested (11), it is possible that metabolic alterations induced by arsenic could start by altering liver physiology. Nevertheless, future studies should address whether short exposures to arsenic can impact insulin signaling in the liver, and affect the metabolism of this organ and its possible involvement with metabolic and endocrine alterations in pancreatic islets, skeletal muscle and adipose tissue.

Additionally, in Wistar rats exposed to arsenic (3 ppm) *in utero* and during early life, developed alteration in the levels of triglycerides, lysophospholipids and phosphatidylcholines (120). These animals had increased levels of lipid peroxidation markers, such as malondialdehyde (120). Noteworthy, oxidative damage on lipids is a common feature of metabolic diseases that predisposes to the development of other conditions such as atherosclerosis and NAFLD (121, 122). Sodium arsenite treatment (100 ppb) *in utero* and until postnatal week 13 exacerbated the effects of a Western diet on the development of NAFLD, induced the overexpression of genes involved in free fatty acid synthesis, triacylglycerol packaging, ketogenesis and beta-oxidation in Swiss Webster male mice (37). Therefore, arsenic exposure during critical developmental windows can produce alterations in lipid metabolism, increasing the risk of chronical diseases during adulthood.

The interaction between diet and arsenic exposure on lipid metabolism has been tested in several studies. Sodium arsenite (25 and 50 ppm for 5 months) decreased triglyceride levels in plasma from NMRI male mice fed with a high-fat diet, without affecting the levels of cholesterol, HDL and LDL (123). Interestingly, there was no effect in the mice receiving a control diet (123). In spontaneous hypertensive prone rats fed with a high-cholesterol diet, early sodium arsenite exposure (133 ppm starting at ten weeks of age) resulted in decreased HDL/LDL ratio, and higher levels of total cholesterol and triglycerides in plasma (124). These works indicate that arsenic can either potentiate or reduce the effects of diet on lipid metabolism, depending on the diet type and ontogeny period of exposure. Future studies will help to clarify under which circumstances arsenic potentiate the effects of hypercaloric diets on lipid metabolism, as well as the impact of these alterations on the development of insulin resistance.

## Arsenic Effects on Cardiovascular Physiology

One of the most common outcomes of MS is the development of CVDs, which are a group of disorders of the macro and microvasculature and the heart. These pathologies include hypertension, thrombosis, peripheral arterial disease, atherosclerosis, heart attack, stroke, heart failure, congenital heart disease, and rheumatic heart disease. Altogether, these conditions are the number one cause of death globally (125, 126). Populations chronically exposed to arsenic in drinking water have an increased risk for developing carotid atherosclerosis, coronary heart disease, peripheral artery disease, and stroke (127–130). Thus, it is interesting to understand whether the cardiovascular alterations induced by arsenic recapitulate the pathophysiological processes observed in MS patients.

The endothelium is a monolayer of endothelial cells localized in the inner wall of blood and lymph vessels (131). Endothelial integrity is fundamental for vascular health because it regulates systemic blood pressure, tissue perfusion, and the recruitment and extravasation of proinflammatory leukocytes in response to tissue damage (131–135). In human microvascular endothelial (HMEC-1) cells, treatment with non-cytotoxic concentrations of sodium arsenite (0.5 and 1 µM for 24 h) reduced the thrombin signaling by decreasing the phosphorylation levels of the endothelial nitrogen oxide synthase (eNOS), lowering NO synthesis and cytosolic Ca^2+^ levels, which could lead to endothelial dysfunction (136). Conversely, treatment with sodium arsenite concentrations above 5 µM during the same time induced cell apoptosis by activating the phospholipase C/inositol-1,4,5-triphosphate (PLC/IP3) signaling, inducing eNOS activity and intracellular Ca^2+^ levels (136). Similarly, treatment with sodium arsenite concentrations above 10 µM induced apoptosis in human umbilical vein endothelial cell (HUVEC) cells (137). In these cell models, apoptosis induction depended on the activation of the EGF, JNK, p38 and p21Cip1/Waf1 signaling pathways (137). The deregulation of NO synthesis (both up- and down-regulation, depending on the arsenic concentration) is interesting because this is a signal messenger that promotes vascular relaxation, but during pathological processes, it generates nitrogen reactive species and oxidative stress, damaging the endothelium (138, 139). Also, alterations in NO signaling and oxidative stress in the endothelium contribute to the development of endothelial insulin resistance, which in turn accelerates the development of hypertension, atherosclerosis, and endothelial dysfunction during the MS (140). It could be interesting to test whether arsenic in fact causes endothelial insulin resistance related to NO deregulation, as this process can affect insulin delivery to other tissues, contributing to muscular insulin resistance (100, 140).

Experiments performed in more complex systems such as *ex vivo* treatment of aortic rings without endothelium from Sprague-Dawley rats (10, 25 and 50 µM) reduced vasorelaxation induced by acetylcholine (141). Thus, indicating that sodium arsenite impairs relaxation of the smooth muscle layer in the blood vessels, which could contribute to hypertension. Concordantly, male Wistar rats treated with 50 ppm of sodium arsenite, or 50 ppm of arsenate for 200 days developed endothelial oxidative stress and hypertension (70). Similarly, male Wistar rats treated with 1.5 mg/kg of sodium arsenite for two weeks developed vascular endothelial dysfunction related to reduced acetylcholine-dependent vascular relaxation, eNOS inhibition, and the closure of the ATP-dependent K^+^ channels (142). Because vascular relaxation is a critical process to maintain blood pressure (143), evidence indicates that arsenic exposure can contribute to hypertension (one of the key characteristics of the MS) by disrupting NO signaling and K^+^ currents in the endothelium and vascular smooth muscle cells.

Atherosclerosis is a chronic inflammatory disease that occurs within the arterial wall; it is characterized by the deposition of oxidized lipoproteins, promoting the differentiation of macrophages into foam cells and forming atheromatous plaques (144). Association studies have shown that arsenic exposure is related to increased carotid intima-media thickness and the presence of carotid plaque, which are markers of atherosclerosis (145–148). Apolipoprotein E-deficient mice (ApoE-/-) treated with 20 or 100 mg/L of sodium arsenite for 24 weeks developed more atherogenic lesions in the aorta than non-treated ApoE-/- mice (149). Interestingly, this effect was independent of the amount of dietary cholesterol, indicating that the mechanisms of arsenic-induced atherosclerosis could be different from those induced by diet (149). Nevertheless, other dietary factors such as polyunsaturated fatty acids, some vitamins and polyphenols can control the progression of this disease in humans (150). Thus, it could be interesting to further address whether arsenic can influence the protective mechanisms induced by these dietary factors during atherosclerosis development.

Mechanistically, primary human aortic endothelial cells treated with sodium arsenite (5 and 50 µM for five hours) did not show increased oxidation of LDL-cholesterol (149). However, these cells had increased levels of IL8, which acts as an endothelial growth factor and a monocyte and lymphocyte chemoattractant during atherosclerosis development (149, 151). Sodium arsenite (5 µM for up to 8 h) induces the overexpression of the antioxidant enzyme heme oxygenase-1 (HO-1) and the cytokines monocyte chemoattractant protein-1 (MCP-1) and IL-6 in human vascular smooth muscle cells (152). The overexpression of these factors resulted in a higher migration of monocytes, suggesting that sodium arsenite can induce monocyte infiltration into atherogenic lesions (152). In HUVEC and ECV304 endothelial cell models, sodium arsenite (up to 30 µM) suppressed the expression of Fas ligand (FasL) due to the activation of ROS-sensitive endothelial cell signaling (153). FasL is an essential factor that prevents macrophages’ extravasation and has an anti-atherogenic activity (154), indicating that another mechanism for sodium arsenite-induced atherosclerosis includes FasL downregulation. As described in the previous section, arsenic also induces proatherogenic dyslipidemias, characterized by low levels of HDL and high levels of LDL cholesterol observed both in epidemiological studies and animal models. Therefore, in combination endothelial dysfunction, the expression of proinflammatory cytokines and monocyte and macrophage recruitment to the vascular walls, it can be concluded that arsenic exposure is a strong factor predisposing to the development of atherosclerosis.

Hyper-coagulability and prothrombotic states are physiological alterations related to MS (155, 156). Thrombosis can be classified as venous thromboembolism and arterial thrombosis (157). Specifically, MS patients have increased plasmatic coagulation, reduced fibrinolysis, hyperreactive platelets, and impaired endothelial thrombo-resistance, increasing the risk for venous and arterial thrombosis (155, 158, 159). Notably, each sign of MS increases the risk of developing venous thromboembolism (155). *In vitro* treatment with up to 50 µM of sodium arsenite in platelets isolated from rat blood resulted in increased aggregation (160). Moreover, sodium arsenite increased the aggregation effect of other compounds involved in blood clot formation, such as collagen, ADP, and arachidonic acid (160). Concordantly, sodium arsenite treatment *in vivo* (10 and 25 ppm in drinking water for four weeks) induced platelet aggregation and arterial thrombosis in rats (160). Infusion of 0.5 mg/kg MMA^III^ in rats resulted in venous thrombosis (19). *In vitro* treatment with a trivalent mono methylated metabolite of arsenic MMA^III^ (monomethylarsonous acid) up to 5 µM for 4 h induces cell apoptosis, phosphatidylserine externalization, caspase activation, and procoagulant activity in human platelets (19). Evidence points out that platelet apoptosis has a vital role in the procoagulatory state and pulmonary thromboembolism (161). Also, hyperlipidemic environments and oxidized LDL, conditions found in MS patients, induce phosphatidylserine externalization, caspase 3 activation, and platelet aggregation (162). Thus, arsenic could potentiate the prothrombotic state characteristic of MS patients by inducing platelet apoptosis. However, this possibility should be addressed in future studies.

Altogether, the evidence supports the participation of arsenic exposure in the development of an array of CVDs related to MS. However, these studies are not focused on the relationship between CVD and metabolic alterations. Future research should address whether arsenic can potentiate the cardiovascular effects of a hypercaloric diet, speeding up the development of alterations of the heart and the macro or microvasculature. Also, the interaction between cardiovascular alterations and the other hallmark of MS needs to be tested to understand the role of arsenic exposure on the development of CVD associated with metabolic abnormalities.

## Arsenic Effects on Inflammation and Immune System

Hypertrophic adipose tissue develops a low-grade chronic inflammation (163–165). In obesity, adipocytes secrete proinflammatory cytokines and adipokines that recruit macrophages and lymphocytes, promoting adipocyte apoptosis, fibrosis, and insulin resistance (39, 164). Moreover, chronic inflammation promotes the development of insulin resistance in muscle and liver and contributes to the development of NAFLD (86, 166, 167). In addition, in obese subjects and T2D patients, the immunosurveillance is compromised, increasing their risk for developing infectious diseases (168, 169). When arsenic is added to the scene, it also dysregulates the immune response, decreasing the response to pathogens and increasing inflammation, as we will discuss.

Exposure to arsenic impacts immune processes, including alterations in the innate and adaptive immune system, decreasing the immune surveillance system, and increasing the rate of infection, autoimmune diseases, cancer, and other immune problems (170–172). In a cohort of Bangladeshi newborns, *in utero* exposure to high arsenic levels correlated with low thymic function and lower levels of signal-joint T-cell receptor-rearrangement excision circles in T lymphocytes (173). Thus, indicating that *in utero* arsenic exposure impairs transplacental immune regulation and could lead to immunosuppression during childhood and adulthood (174). Another study performed in adults from Bangladesh reported that arsenic exposure levels correlated with higher serum abundance of Th2 cytokines and asthma (175). Populations exposed to arsenic also have impaired T cell proliferation induced by concanavalin A and lower secretion of T cell-derived cytokines, such as IL-2, IFN-ϒ, and TNFα (176). These population-based studies indicate that arsenic can promote immunosuppression. Nevertheless, it remains to be determined whether arsenic could potentiate the immunocompromised state of MS and T2D patients.

Contrarily to the immunosuppressor effects of arsenic, chronic exposure to this pollutant can induce chronic inflammation, oxidative stress, and upregulation of TNFα and IL-6; related to liver damage, the development of CVDs, increased risk to develop malignancies, and Alzheimer’s disease (177–180). For example, in a Taiwanese population exposed to different concentrations of arsenic through drinking water, the lymphocytes had increased expression of several proinflammatory cytokines such as IL-1β, IL-6, chemokine C-C motif ligand 2/monocyte chemotactic protein-1 (CCL2/MCP1), chemokine C-X-C motif ligand 1/growth-related oncogene alpha, and the chemokine C-X-C motif ligand 2/growth-related oncogene beta (181). Nevertheless, the association between arsenic, inflammation markers, insulin resistance, and MS remains unexplored in epidemiologic studies.

In experimental models, the effects of arsenic on the immune system depend on age, species, and the dose and time of exposure. In pregnant Balb/c [H-2^d^] mice treated with 4 ppm of arsenic trioxide from the time of conception until parturition, the offspring exhibited low levels of IgG_2a_, reduced allogeneic stimulation of CD4^+^ T cells, reduced number of splenic CD4^+^ and CD8^+^ lymphocytes, and a higher susceptibility to *Escherichia coli* infection (182). All these signs indicate that prenatal exposure to environmental arsenic can have long-lasting effects on the offspring’s immune system, even in the absence of further exposure. Infection with AH1N1 influenza virus in male C57BL/6J mice previously exposed to sodium arsenite (100 ppb for five weeks) resulted in higher morbidity, higher levels of CD8^+^ in the bronchoalveolar lavage fluid, and decreased number of dendritic cells in the mediastinal lymph nodes (172). Since obesity and insulin resistance are associated with lower immune responses and chronic inflammation, it will be interesting to test the possible interaction between arsenic and diet-induced obesity in animal models.

*In vitro* treatment of lymphocytes from healthy donors with sodium arsenite (up to 1 µM for 48 and 72 h) showed that this metalloid is more toxic to T helper (Th) than to cytotoxic T lymphocytes (Tc) (183). In murine and human lymphocytes treated with 1 µM of sodium arsenite, the cells produced less IL-2 and had impaired proliferation when stimulated by phytohemagglutinin (184, 185). In human lymphocytes, treatment with sodium arsenite (up to 1 µM for 48 h) increases intracellular Ca^2+^ concentrations induced by mitogenic stimulation (186). This later effect correlated with reduced proliferation in response to the mitogenic stimulus and increased cell apoptosis (186).

Glucose metabolism and glucose uptake are necessary to maintain proper lymphocyte function and to sustain proliferation in the presence of immunogenic stimulus (187–190). Also, the lymphocytes from T2D patients had reduced glucose metabolism, the expression of the GLUT transporters, and the activity of calpains, which are proteases involved in regulating GLUTs (191–194). *In vitro* treatment of human lymphocytes with up to 1 µM of sodium arsenite for 72 h resulted in reduced GLUT1 trafficking, alterations in glucose uptake and calpain activity (195). Thus, indicating that alterations induced by arsenic on the glucose metabolism of lymphocytes recapitulate some of the characteristics observed on lymphocytes from T2D patients. Interestingly, arsenic can promote calpain activation in cell-free systems, indicating that calpain proteases could be a direct target for arsenic-induced metabolic and immune alterations (195). Altogether, the evidence supports the concept that arsenic exposure can impair lymphocyte functions, resulting in immunocompromised states and reduced immunosurveillance. Nevertheless, future research should address whether hypercaloric diets and obesity could potentiate the immunotoxic effects of arsenic on lymphocytes.

Macrophages are an innate immune effector cell type (196). This cell lineage has several functions regulating the physiology of metabolic-relevant tissues, such as the endothelium, liver and adipose tissues (196, 197). In RAW 264.7 murine and THP1 human macrophage cell lines, treatment with arsenic trioxide resulted in decreased expression of target genes of the liver X receptor/retinoid X receptor (LXR/RXR) transcription factor complex, leading to reduced cholesterol efflux (198), promoting the development of foam cells and atherogenic plaques.

Macrophages can be induced to differentiate into classically activated M1 and alternatively activated M2 macrophages (196). These activated macrophages play essential roles in developing MS, such as insulin resistance in the adipose tissue, NAFLD, and liver fibrosis (196, 199). In primary cultures of human macrophages, treatment with arsenic trioxide (1 µM for 72 h) decreased some human macrophage-specific genes interfering with their differentiation program (200). The alterations in macrophage gene transcription are dependent on redox-sensitive signaling pathways, such as the transcription factors Nrf2, Bach1 and EGR2, which are essential for macrophage differentiation and function (201).

Kupffer cells are liver-residing macrophages that play important roles in developing of hepatic insulin resistance and fibrosis induced by diet (202, 203). *In vitro* treatment of THP-1 derived macrophages with sodium arsenite (up to 8 µM for 48 h) promoted differentiation into M2 macrophages, inducing secretion of fibrogenic cytokines *via* upregulation of the miRNA miR-21 (204). This pathway correlated with the observation in the liver from C57BL6 mice exposed to 40 ppm of sodium arsenite for six months. In the liver of these mice, sodium arsenite promoted M2 differentiation, miR-21 overexpression, and liver fibrosis (204). These effects were prevented in miR-21 KO mice, highlighting the relevance of this mechanism (204). Concordantly, *in vivo* inhibition of Kupffer cells with gadolinium chloride prior treatment with 1.5 mg/kg/day of sodium arsenite for 28 days in Wistar rats improved liver function, serum lipid profile and restored the cell architecture of the liver (68). Highlighting the important role of macrophages in liver dysfunction. However, the effects of arsenic exposure along with high-fructose or high-fat diets, which promote liver damage (205) are unknown. The alterations induced by arsenic on the macrophages that reside in the adipose tissue, which are essential in modulating insulin sensitivity and chronic inflammation during obesity (197) are unknown. These questions will be relevant to understand the role of macrophages in arsenic-induced metabolic alterations.

## The Effects of Arsenic on Ionic Channel Function as a Mechanism for Arsenic-Induced Metabolic Alterations

Ionic channels are complex transmembrane macromolecules forming aqueous pores, which once activated constitute selective functional pathways for ions to move through by diffusion down their electrochemical gradients (206). These proteins have fundamental roles in insulin secretion, insulin signaling, cardiovascular health, and the immune system (45, 207–209). This section reviews two possible mechanisms for arsenic-induced ion channel alteration, (i) as a solute permeating bidirectionally through aquaglyceroporins, (ii) exerting a direct toxic effect by inhibiting the functional activity of different ion channels.

Aquaglyceroporins (AQP) are transmembrane channels permeable to water, glycerol, urea, and other small non-charged or bipolar solutes (210, 211). AQP7 and AQP9 are mainly expressed in the adipose tissue and the liver, respectively (212). In these tissues, AQP7 and AQP9 participate in glycerol transport and participate in the development of insulin resistance (212, 213). Also, AQP7 is expressed in normal and tumoral pancreatic β-cells, and the existing data suggest that it plays a role in GSIS (214, 215).

Since at physiological pH sodium arsenite adopts the form As(OH)_3_, resembling glycerol structure, it is known that AQP7/9 facilitates sodium arsenite uptake by the cells (216, 217). This fact raises two possibilities: 1) AQP7/9 increases arsenic uptake into adipocytes, hepatocytes, and pancreatic β-cells, increasing the toxic effects on these cell types. 2) Sodium arsenite could interfere with the normal functions of these channels, altering metabolic processes in the adipose tissue, liver and pancreatic islets. However, these possibilities should be systematically addressed in future studies. Contrarily, AQPs are involved in arsenite detoxification pathways by promoting its excretion (218, 219). Thus, it will be relevant to study the possible effects of arsenic interaction with these channels during the development of metabolic alterations.

Arsenic compounds affect protein structure and function by directly interacting with these macromolecules (17). Nevertheless, the protein-arsenic interactions between ion channels remain poorly characterized. The voltage-dependent anion channel (VDAC) is an integral constituent of the mitochondrial outer membrane permeability transition pore (PTP) complex (220). This channel is a target of arsenic trioxide (up to 5 µM for 30 min) in liver mitochondria, resulting in cytochrome c release and decreased mitochondrial membrane potential (221). The relevance of this mechanism was highlighted by the fact that cytochrome c release was prevented when the mitochondria were treated with an antibody against this channel (221). Potentially, this mechanism could contribute to apoptosis and liver inflammation induced by arsenic, as we reviewed in previous sections. Also, arsenic trioxide, in combination with ascorbic acid and disulfiram, inhibits VDAC *via* the generation of ROS in five different pancreatic cancer cell lines, leading to proposed VDAC-mediated necrosis (222). This latter process could be especially relevant in pancreatic islet degeneration which was revised in section 3.

In lymphocytes from humans chronically exposed to arsenic in China, there was a downregulation on the expression of KCNE1, KcNH2, KCNQ1, and CACNA1C genes (223). These code for subunits of the Kv β, HERG/Kv11.1, Kv7.1, and L-type Cav2.1 ion channels, respectively (223). The cardiomyocyte cell line AC16 treated with 5 µM sodium arsenite for 72 h showed the same results (223). Thus, indicating that arsenic could exert some of its immune and cardiovascular toxic effects through deregulating ion channels in different cell lineages.

The use of arsenic trioxide as an antineoplastic drug for treating acute promyelocytic leukemia is linked with severe cardiotoxicity (224). Specifically, several studies describe those patients treated with therapeutic doses of arsenic trioxide develop cardiac arrhythmia (225–229). This form of arsenic increases cardiac calcium currents and decreases human Ether-à-go-go-Related Gene (hERG) channel expression, trafficking to the plasma membrane and potassium current (230–232). These effects of arsenic trioxide on cardiac ionic currents result in elongating the QT interval of the electrocardiogram by delaying the repolarization phase (230, 233). On the other hand, exposure of fibroblast L cell line overexpressing the KCNA5 gene (encoding the hKv1.5, a cardiac potassium channel) resulted in increased current density (230). Therefore, evidence points out ionic channels as important targets during the development of cardiac diseases induced by arsenic.

Since GSIS by pancreatic beta-cells is regulated by the orchestrated opening and closure of different ion channels (51), it is tempting to conceive that these proteins could play an important role in developing MS and T2D. However, the evidence so far has only shown that arsenic can decrease intracellular calcium (iCa^2+^) levels (73), without directly addressing the effects of this pollutant on any of the channels involved in iCa^2+^ concentrations. Future research could help enlighten these processes.

## Unifying Mechanisms Across Tissues

Comparing the available information about the multiple effects of arsenic on processes that could be involved in MS pathogenesis is challenging due to several aspects of experimental design used by different research groups (**Table 1**). For instance, some studies apply arsenic through drinking water, in animal studies, while others give the arsenic by gavage. Also, there is no consensus about the arsenic dose and exposure time. These limitations need to be overcome to correctly draw a precise picture of the physiopathology of MS induced by arsenic. The main discrepancies that we observed across the literature were about which is the first metabolic abnormality induced by arsenic. While some works reported insulin resistance and adipose tissue dysfunction, other authors indicate that pancreatic beta-cell dysfunction is the main target of arsenic exposure. Noteworthy, rat models tend to develop many aspects related to MS when treated with arsenic, including insulin resistance, proatherogenic dyslipidemia, non-alcoholic liver steatosis, heart damage and hypertension, while mouse models seem to develop glucose intolerance and beta cell failure, without developing dyslipidemia and insulin resistance (**Table 1**). Based on the available evidence, we propose that rat models better recapitulate the alterations related to MS induced by arsenic that could happen in human populations exposed to this pollutant.

We can propose a general model based on the results from three studies performed with comparable arsenite doses (ranging from 2.5 to 5 mg/kg/day of sodium arsenite by oral gavage) for 3 or 6 months in male rats. These studies report that at 3 months of exposure, the main effects of arsenite are insulin resistance, glucose intolerance and hyperinsulinemia (46, 53, 111). In contrast, after 6 months of exposure, pancreatic beta-cell mass is lost through ferroptosis programmed cell death (65). As the methods employed in these studies are very similar, it is tempting to think that during short exposures to arsenic, the first alterations in whole-body glucose homeostasis are related to insulin resistance, rather than beta-cell dysfunction and loss. In contrast, a longer exposure time results in beta-cell loss and reduced plasma insulin levels. This model is in accordance with the prevailing view that insulin resistance precedes impaired GSIS and beta-cell exhaustion during MS pathogenesis (2). However, it is important to note that some other works propose that at short exposure times, one of the primary targets of arsenic is pancreatic beta-cell dysfunction, not insulin resistance (61). Perhaps these different modes of action could be related to sex dimorphism, arsenic dose, species and development window of exposure? Arsenic could affect glucose homeostasis through different pathways depending on the context of the case. For instance, some modes of action could target insulin-responsive tissues, while under other circumstances, beta cell dysfunction occurs first, triggering T2D without necessarily inducing the other signs of MS. These different models could also explain the discrepancies between epidemiological studies aimed at determining the association between arsenic exposure with insulin resistance and beta-cell function across different populations.

In the context of insulin resistance, an unanswered question is whether different insulin target tissues have different sensitivity to arsenic exposure. For instance, the liver is the main organ involved in arsenic metabolism (18), and due to its direct connection to portal circulation (which transports most of the arsenic ingested through drinking water (18)) it could be more sensitive to the endocrine and metabolic disrupting effects of arsenic and its methylated products. Concordantly, 28 days of arsenite treatment result in Kupffer cell activation in the liver, causing liver damage and dyslipidemia (68). Indicating that metabolic impairment and insulin resistance in liver could precede the defects in skeletal muscle and adipose tissues. Therefore, it will be interesting to test in future studies the possibility that short-term arsenic exposure could affect liver metabolism and insulin sensitivity *in vivo*, before affecting muscular and adipose tissue insulin signaling. Also, it will be interesting to test whether arsenic can impair insulin signaling through pathways other than Akt phosphorylation in the liver, skeletal muscle, and adipose tissues. This will permit stablishing a more detailed model of the endocrine-disrupting actions of arsenic on insulin-regulated processes.

Regarding the effects of arsenic and obesity, most of the evidence indicates that arsenic’s metabolic and endocrine effects are independent of weight gain and increased adiposity. Nevertheless, *in vitro* and *in vivo* evidence points to the notion that arsenic induces adipocyte hypertrophy, adipose tissue metabolic dysfunction, and alterations in adipokine secretion (41, 42, 99). Thus, even without increasing adiposity, arsenic can affect the functions of adipose tissue, mimicking alterations associated with obesity and MS. This point is of particular interest, as there is increasing evidence that around 20% of the average weight adult population have a higher risk of developing cardiovascular diseases, due to unhealthy metabolic parameters (234, 235). Some authors hypothesize that these metabolically unhealthy lean subjects could have a lipodystrophy-like phenotype, resulting in a higher risk to develop NAFLD, insulin resistance and impaired insulin secretion (235). It is tempting to think that arsenic exposure could induce similar alterations in adipose tissue, increasing the risk of developing the other signs of MS. This idea should be further explored in forthcoming studies.

One of the most studied outcomes of arsenic exposure is cardiovascular alterations. Mainly, we summarized evidence that arsenic impairs the relaxation of blood vessels, causing hypertension after two weeks of treatment (70, 142). On the other hand, insulin induces vasodilation through NO synthesis, and evidence indicates that insulin resistance in the endothelium could link MS and hypertension (236). Thus, one possibility is that endothelium insulin resistance could arise even after short arsenic exposures.

Finally, local inflammatory activation of immune cells has a pivotal role in developing insulin resistance in the liver, adipose tissue and muscle (100, 165, 237). Chronic inflammation during MS is also implicated in NAFLD development, adipocyte dysfunction, muscle remodeling, atherosclerosis development and damage to the endothelium and the heart (238). As mentioned above, arsenic can trigger Kupffer cells’ activation towards the inflammatory phenotype in the liver (68). It can also promote cholesterol uptake in macrophages, promoting atherosclerosis development (198). Indicating that arsenic could exert some of its metabolic disrupting functions by promoting local inflammatory processes. However, there is a lack of information about the effects of arsenic in immune cells residing in the adipose tissue and muscle.

## Knowledge Gaps and Future Directions

One of the key characteristics of T2D is beta-cell dysfunction, reducing plasma insulin concentration, which combined with insulin resistance results in hyperglycemia (239). In this regard, most of the available evidence for the metabolic impact of arsenic focus on its diabetogenic effects through beta-cell dysfunction and impaired GSIS. Still, MS is a condition preceding T2D, increasing the risk of developing several pathologies. Future research needs to test the effects of arsenic on processes related to MS that occur prior to dysfunction and loss of pancreatic beta-cells. Hence, it is necessary to explore lower doses of arsenic for shorter exposure times to understand the arsenic effects when pancreatic beta-cells are relatively intact. This strategy will allow us to recapitulate the progression of alterations that lead to T2D.

Moreover, some of the signs of MS can be reversed through modification in diet and lifestyle (240, 241). At this moment, it is unknown whether the effects of arsenic on the signs related to MS can be mitigated or reversed by limiting arsenic exposure, or dietary interventions and exercise. Future works should address these questions *in vitro* and *in vivo* models of arsenic exposure.

The most studied risk factors for developing MS are hypercaloric diets and sedentary lifestyles. There is probable an interaction of these factors with arsenic in exposed populations. Experimental models should test the effects of arsenic consumption with different diet regimens and different degrees of physical activity. The analysis of these possible interactions will lead to a deeper understanding of arsenic’s endocrine and metabolic disruption activity during the development of MS.

Finally, there is a need for studies addressing whether the alterations in the different organs and tissues described in animal models recapitulate the alterations induced by humans environmentally exposed to arsenic. For instance, rodents have a lower sensitivity to arsenic due to the differences discussed previously and, therefore, the doses used in experimental animal models are orders of magnitude higher than the doses to which humans are exposed (**Table 1**). For this reason, studies using biopsies of the different tissues will help understand how the processes that lead to MS are affected in humans and if data extrapolation between species is of clinical relevance.

## Concluding Remarks

Arsenic pollution represents an important health risk for millions of people worldwide. This pollutant can deregulate the metabolism of the entire organism, promoting the development of obesity, insulin resistance, dyslipidemia, hypertension, atherosclerosis, NAFLD while impairing insulin secretion. This array of alterations can potentially contribute to the development of MS, and even accelerate the progression of this pathology. Nevertheless, there are few works that evaluate the metabolic syndrome in exposed humans and animal models. To better understand the development of metabolic syndrome induced by arsenic, future research should address its interaction with hypercaloric diets and the development of the hallmark signs of metabolic syndrome. Based upon the preponderance of the diverse and complementary data currently available, we conclude arsenic should be considered a risk factor for the metabolic syndrome and its associated comorbidities.

## Author Contributions

PP: Conceptualization, research, writing draft. MV: research, writing draft. AS: research, writing draft. AP: research, writing draft. RO-H: figures preparation, writing draft. GG-P: research, writing draft. MS-B: research, writing draft. PO-W: research, writing draft, edition of final manuscript, funding acquisition. MH: Conceptualization, research, writing draft, edition of final manuscript, funding acquisition. All authors contributed to the article and approved the submitted version.

## Funding

This work was supported by CONACYT-Fronteras grant #568492 and PAPIIT-UNAM grant IN208720.

## Conflict of Interest

The authors declare that the research was conducted in the absence of any commercial or financial relationships that could be construed as a potential conflict of interest.

## Publisher’s Note

All claims expressed in this article are solely those of the authors and do not necessarily represent those of their affiliated organizations, or those of the publisher, the editors and the reviewers. Any product that may be evaluated in this article, or claim that may be made by its manufacturer, is not guaranteed or endorsed by the publisher.
